# Modified transport medium for improving influenza virus detection

**DOI:** 10.3389/fcimb.2024.1399782

**Published:** 2024-07-04

**Authors:** Zhiqi Zeng, Qianying Li, Hua Guo, Yong Liu, Lixi Liang, Yuanfang Lai, Yi Fang, Lei Li, Qiuting Xue, Yangqing Zhan, Zhengshi Lin, Wenda Guan, Zifeng Yang

**Affiliations:** ^1^ Guangzhou Key Laboratory for Clinical Rapid Diagnosis and Early Warning of Infectious Diseases, KingMed School of Laboratory Medicine, Guangzhou Medical University, Guangzhou, China; ^2^ Respiratory Disease AI Laboratory on Epidemic Intelligence and Medical Big Data Instrument Applications, Faculty of Innovative Engineering, Macau University of Science and Technology, Macao, Macao SAR, China; ^3^ State Key Laboratory of Respiratory Disease, National Clinical Research Center for Respiratory Disease, Guangzhou Institute of Respiratory Health, The First Affiliated Hospital of Guangzhou Medical University, Guangzhou, Guangdong, China; ^4^ BaSO Diagnostics Inc., Zhuhai, Guangzhou, China; ^5^ Kingmed Virology Diagnostic and Translational Center, Guangzhou Kingmed Center for Clinical Laboratory Co., Ltd., Guangzhou, China; ^6^ Guangzhou Laboratory, Guangzhou, Guangdong, China

**Keywords:** influenza, modified transport medium (MTM), viral load, diagnosis, sensitivity

## Abstract

**Background:**

Accurate detection of influenza virus in clinical samples requires correct execution of all aspects of the detection test. If the viral load in a sample is below the detection limit, a false negative result may be obtained. To overcome this issue, we developed a modified transport medium (MTM) for clinical sample transportation to increase viral detection sensitivity.

**Method:**

We first validated the MTM using laboratory-stocked influenza A viruses (IAVs: H1N1, H3N2, H7N3, H9N2) and influenza B viruses (IBVs: Yamagata, Victoria). We also tested clinical samples. A total of 110 patients were enrolled and a pair of samples were collected to determine the sensitivity of real-time polymerase chain reaction (RT-PCR) following MTM treatment.

**Result:**

After 24 h culturing in MTM, the viral loads were increased, represented by a 10-fold increase in detection sensitivity for H1N1, H9N2, and IBVs, a 100-fold increase for H3N2, and a 1,000-fold increase for H7N3. We further tested the effects of MTM on 19 IAV and 11 IBV stored clinical samples. The RT-PCR results showed that the positive detection rate of IAV samples increased from 63.16% (12/19) without MTM culturing to 78.95% (15/19) after 48 h culturing, and finally 89.47% (17/19) after 72 h culturing. MTM treatment of IBV clinical samples also increased the positive detection rate from 36.36% (4/11, 0 h) to 63.64% (7/11, 48 h) to 72.73% (8/11, 72 h). For clinical samples detected by RT-PCR, MTM outperformed other transport mediums in terms of viral detection rate (11.81% increase, P=0.007).

**Conclusion:**

Our results demonstrated that the use of MTM for clinical applications can increase detection sensitivity, thus facilitating the accurate diagnosis of influenza infection.

## 1 Introduction

Acute respiratory tract infection (ARTI) is one of the most common infectious diseases in humans worldwide. ARTI is generally caused by bacteria and viruses, with approximately 80% of ARTIs being respiratory viral infections (Zhang et al., 2020). Respiratory tract infections are more common in children and older adults, and the disease progresses more rapidly in immunocompromised individuals, leading to serious illness and death. In 2008, approximately 109,500,000 influenza virus cases occurred globally, with 15,300 hospital deaths of children under 5 years of age, imposing a heavy burden on healthcare systems worldwide (Wang et al., 2020). Therefore, early and accurate detection of influenza viruses is important to save lives and control outbreaks. However, current tests often yield false-negative results because of the sample extraction, transport, storage, and testing processes. The problems encountered during these processes include: 1) substandard specimen collection, 2) improper transport leading to degradation of viral nucleic acids, and 3) low viral loads in patients and insufficient kit sensitivity. Therefore, proper storage and transport of respiratory specimens are important in laboratory detection of influenza virus infections (Wu et al., 2021).

Various viral transport media are commercially available for better viral transport and preservation. These include both inactivating and non-inactivating transport media. Inactivating transport media contain high concentrations of lysis salts that inactivate the viral proteins in a sample and are generally used to detect viral nucleic acids. Non-inactivating transport media contain a balanced salt solution to maintain viral activity and a source of protein for viral particle stability (Welch et al., 2020). The latter is generally used for viral cultures and antigen detection. Charcoal viral transport medium (CVTM) is specifically designed for the transportation of adenovirus, coxsackie virus, and herpes viruses at room temperature (Leibovitz, 1969; Jensen and Johnson, 1994). The BD Universal Viral Transport System (UVT) has been designed to efficiently maintain HSV-1, influenza A, and RSV (Dsa and Kadni TS, 2023). Additionally, PrimeStore^®^ MTM, an inactivated transport medium, effectively preserves the influenza A virus strain A/California/04/2009 at a temperature of 25°C for a duration of 30 days (Schlaudecker et al., 2014). Because of the lack of viral transmission media (VTM) during the COVID-19 pandemic, several components of transmission mediums have been developed for virus transmission, culture separation, and molecular diagnostics (McAuley et al., 2021; Mears et al., 2021). However, these commercially available transport media do not increase sensitivity or provide effective support for accurate testing of samples with low viral loads. Therefore, we developed a modified transport medium (MTM) to improve the sensitivity of influenza virus molecular detection for more accurate diagnosis. This product is the first transport medium to incorporate culture cells. Regarding the biosafety of MTM, a transfer tube made of polypropylene is employed, which is highly robust and resistant to breakage. Prior to testing, the tube can be injected with inactivator to effectively prevent infection in individuals.

## 2 Methods

### 2.1 Ethical statement

The clinic trial and the use of frozen clinical specimens for evaluation of MTM was approved by the Ethics Committee of The First Affiliated Hospital of Guangzhou Medical University, China, with the project identification code EC-2022049 (SJ)-01.

### 2.2 Development of the modified transport medium

**Figure 1:**
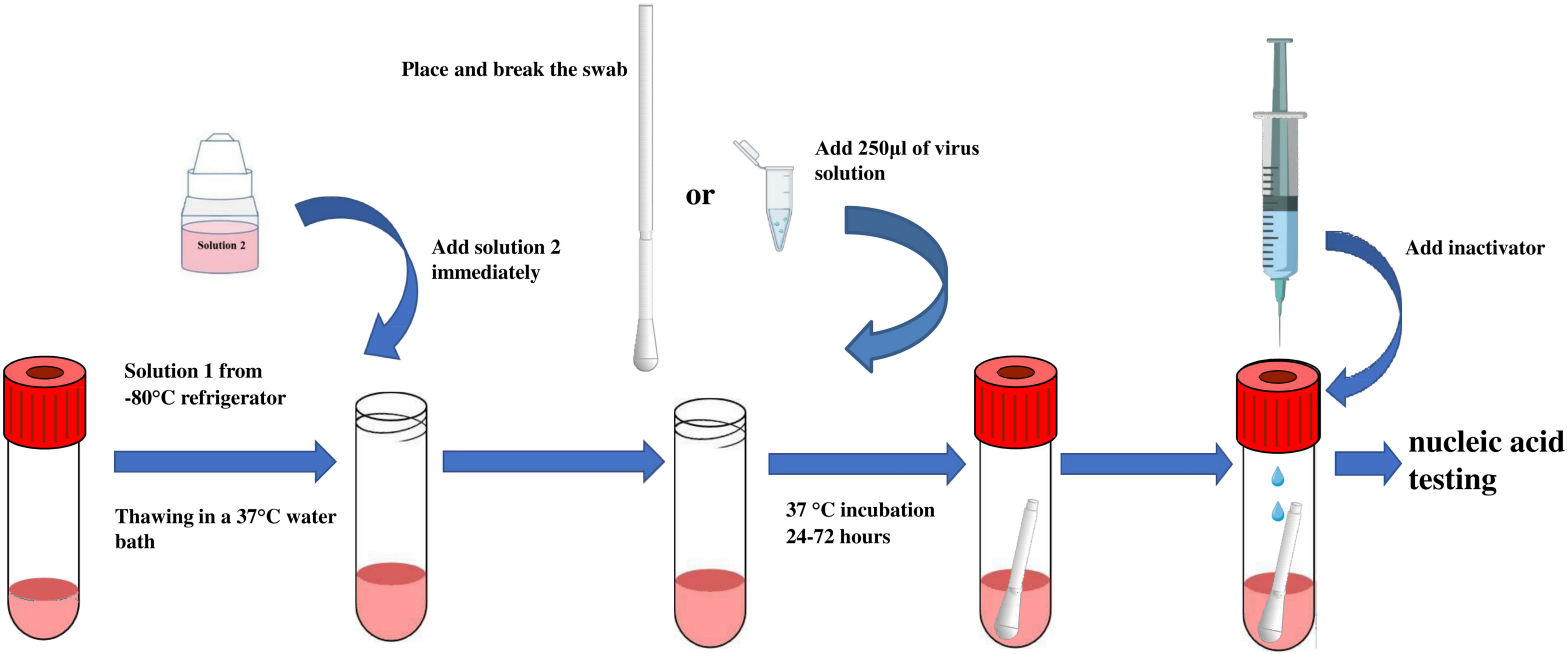
Schematic illustration of MTM application. MTM solution 1 is rapidly thawed by removing them from the −80°C freezer and placing them in a 37°C water bath with the liquid in the tube completely submerged. Immediately after melting, solution 2 is added (pre-equilibrated to room temperature) and mixed with gentle shaking to form working MTM. Next, a respiratory influenza pharyngeal swab is placed in MTM and incubated at 36.5°C ± 0.5°C for 24–72 hours.

The MTM consists of Solution 1 and Solution 2. Solution 1 contains influenza virus-sensitive Madin–Darby canine kidney (MDCK) cells (CCL-34; American Type Culture Collection, Manassas, VA, USA) and Liquid A, a solution consisting of serum-free medium (Baso, Zhuhai, China) and 5% dimethyl sulfoxide (Xilong Science, Shenzhen, China). Solution 1 is stored at −80°C prior to use. Solution 2 consists of 2 μg/ml L-1-tosylamido-2-phenylethyl chloromethyl ketone (TPCK) trypsin (Sigma, Germany) and serum-free medium (Baso, Zhuhai, China) and is stored at 4°C prior to use (**Table 1**). **Figure 1** depicts the preparation process of MTM: Solution 1 is rapidly thawed in a 37°C water bath, then immediately after melting, Solution 2 is added (equilibrated to room temperature) and mixed by gentle shaking to form working MTM. Pharyngeal swab samples were incubated in the medium at 36.5°C ± 0.5°C for 24–72 h.

**Table 1:** Components of the modified transport medium.

	Component	source	Storage temperature
Solution 1	MDCK cell	American Type Culture Collection	-80°C storage
	Serum-free medium	Baso, Zhuhai, China	
	5% dimethyl sulfoxide	Xilong Science, Shenzhen, China	
Solution 2	2 μg/ml TPCK trypsin	Sigma, Germany	4°C storage
	Serum-free medium	Baso, Zhuhai, China	

### 2.3 Evaluation of RT-PCR of influenza test strains treated with MTM

Influenza A viruses H1N1 (A/Guangzhou/GIRD07/2009), H3N2 (A/Aichi/2/1968), H7N3 (A/Duck/Zhejiang/690/2009 [H7N3] [ZJ690]), and H9N2 (A/HK/Y280/97 [A/H9N2]), and influenza B viruses Yamagata (B/Guangzhou/12/2016 [Yamagata-like, Y12]) and Victoria (B/Guangzhou/0215/2012 [Victoria-like, V0215]) were tested. Using the Reed–Muench method, the viral titer was initially determined by the 50% tissue culture infectious dose (TCID_50_) based on the cytopathic effect. Each virus subtype stock solution was 10-fold serially diluted with Dulbecco's modified Eagle's medium (DMEM). Each diluted viral solution was tested using real-time polymerase chain reaction (RT-PCR) with three replicate wells per dilution. The lowest titer of each diluted virus solution detectable by the influenza virus nucleic acid test kit was determined.

Next, 250 µl of each viral dilution of different influenza virus subtypes was added to MTM and incubated at 37°C for 0, 24, 48 and 72 h, respectively, with three replicate wells per titer at each time point. As a comparison, each influenza virus subtype at 100 TCID_50_ and 10 TCID_50_ (H9N2) was treated with a commercial transport medium in the same way.

Throat swab samples were collected from patients with influenza infection and were stored at 2°C–8°C in the commercial viral transport medium and transported to the laboratory, following which they were either immediately tested or stored at −80°C until further testing. SD inactivated lysate (250 μl; Baso, Haizhu, China) was added to each cryopreservation tube prior to nucleic acid extraction. RNA was extracted from the samples using the FastPure Viral DNA/RNA Mini Kit (Vazyme, Nanjing, China) according to the manufacturer's protocol. RT-PCR assays were performed using the influenza A and influenza B TaqMan RT-PCR testing kits (Guangzhou HuYanSuo Medical Technology Co., Ltd, Guangzhou, China) (Inf. A Cat no: HYSS0J02; Inf. B Cat no: HYSS0J03), as previously reported (Zeng et al., 2018). A negative and positive control were also set up and a CT value ≤35 was considered positive.

### 2.4 Evaluation by RT-PCR of influenza clinical samples treated with MTM

In total, 19 influenza A and 11 influenza B samples, which had been collected and stored at −80°C for more than 1 year, were cultured for 48 and 72 h in MTM and tested using the TaqMan RT-PCR testing kit.

### 2.5 Clinical performance

The study was conducted between March 13 and 17, 2023, at Guangzhou Medical University's First Affiliated Hospital and included participants with respiratory infection symptoms. The inclusion criteria were as follows: (i) individuals with acute symptoms such as fever, headache, myalgia, rhinorrhea, nasal congestion, or cough; (ii) individuals who had not taken antiviral medication within 48–72 h of disease onset; (iii) individuals who did not experience severe discomfort during pharyngeal swab sampling; and (iv) individuals who willingly consented to participate in this study and provided written informed consent. The study included 110 individuals who presented with symptoms consistent with respiratory tract infections. Each patient provided two pharyngeal swab specimens, which were then placed in MTM and viral transport medium (Youkang, Beijing, China). MTM samples were cultivated at 36.5°C ± 0.5°C for 48 h and inactivated at 56°C for 60 min. Then, 200 µl of culture was used for nucleic acid extraction using a nucleic acid purification reagent (Biocomma, Shenzhen, China). The extracted nucleic acid was then detected using RT-PCR using the Influenza A & B Virus Nucleic Acid Assay Kit provided by Liferiver, Shanghai Zhijiang Biotechnology Co. Ltd. Furthermore, Youkang transport medium was used for nucleic acid extraction and detection using the same reagent without culture. A cycle threshold (CT) value ≤42 for the FAM channel indicated positive detection of influenza A virus, while a value ≤42 for the VIC channel indicated positive detection of influenza B virus.

### 2.6 Data analysis

The cycle threshold (CT) values of the respiratory viruses are expressed as the mean ± standard deviation using GraphPad Prism (version 8.0.1; GraphPad Software, San Diego, CA, USA). Group comparisons were conducted using t-tests and McNemar's analyses. The sensitivity, specificity, likelihood ratio, positive predictive value, and negative predictive value were analyzed using VassarStats (http://vassarstats.net). A *P* value < 0.05 was considered statistically significant.

## 3 Results

### 3.1 Performance of the test strains with MTM treatment

**Figure 2:**
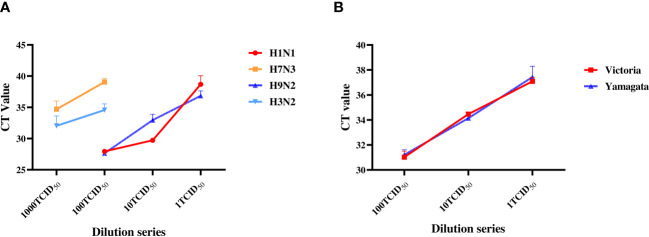
Sensitivity determination with RT-PCR on the laboratory stock IAVs and IBVs. Stock viruses were tenfold serially diluted with DMEM: H3N2 and H7N3 were diluted from 1,000 to 1 TCID_50_; H1N1, H9N2, Yamagata and Victoria were diluted from 100 TCID_50_ to 1 TCID_50_. RT-PCR was then performed separately to determine the minimum detection titer.

**Figure 3:**
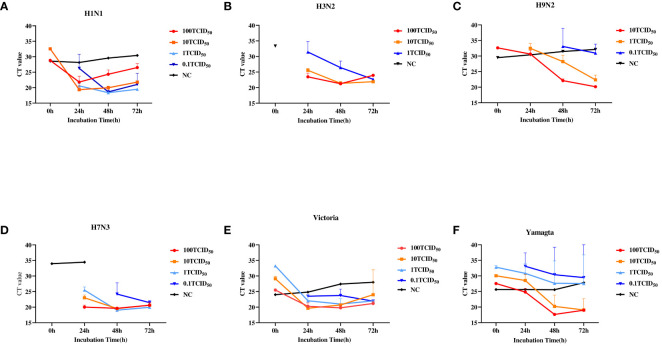
Relationship between RT-PCR cycle threshold (CT) values of IAVs and IBVs and culturing conditions. Viral stock solutions of H1N1, H3N2, H7N3, H9N2, Yamagata, and Victoria were diluted with DMEM to 100, 10, 1, and 0.1 TCID_50_. Then, 250 µl of each diluted viral solution was added to MTM and incubated at 37°C for 0, 24, 48, and 72 hours, with three replicate wells per titer at each time point. As a control (NC), 100 TCID_50_ for H1N1, H3N2, H7N3, Victoria, and Yamagata and 10TCID_50_ for H9N2 were cultured in a commercial medium for 24, 48, and 72 hours. **(A)**, H1N1. **(B)**, H3N2. **(C)**, H9N2. **(D)**, H7N3. **(E)**, Victoria. **(F)**, Yamagata. TCID_50_, 50% tissue culture infectious dose. A CT value >35 was considered negative according to manufacturer's product description.

We first determined the minimal detection sensitivity of RT-PCR using the stocked IAVs and IBVs. Viral stocks were 10-fold serially diluted before the RT-PCR assay. The minimal detection sensitivities were 1 TCID_50_ for H1N1 and H9N2, and 100 TCID_50_ for H3N2 and H7N3 (**Figure 2A**). The minimal detection sensitivity for both IBV Yamagata and Victoria sublineages was 1 TCID_50_ (**Figure 2B**, **Table 2**). We then compared the detection sensitivity of the stock viruses before and after incubation in MTM with the same RT-PCR testing kits (**Table 3**). Viral nucleic acid of H1N1 was undetectable at 1 TCID_50_ before MTM treatment but after 24 and 48 h incubation in MTM with mean CT values of 20.60 (*P*=0.0004) and 18.38 (*P*<0.0001), respectively, both showing statistical significance (**Figure 3A**). The same effects were observed for other viruses treated with MTM: H3N2 virus changed from undetectable at 100 TCID_50_ to detectable with a mean CT value of 23.47 after 24 h incubation (*P*=0.0019) and 21.25 after 48 h incubation (*P*=0.0001, **Figure 3B**); H9N2 virus changed from undetectable at 1 TCID_50_ to detectable with a mean CT value of 32.49 after 24 h incubation (**Figure 3C**) with statistically significant (*P*=0.0184). At 0.1 TCID_50_, H9N2 virus changed to positive after 48 h incubation with a mean CT value of 33.10, but this was not statistically significant (*P*=0.1504); H7N3 virus changed from undetectable at 100 TCID_50_ to detectable with a mean CT value of 20.00 after 24 h incubation (*P*=0.0004) and 19.64 after 48 h incubation (*P*=0.0001, **Figure 3D**); IBV Victoria virus changed from negative at 0.1 TCID_50_ to detectable with a mean CT value of 23.51 after 24 h incubation (*P*=0.0001) and 23.73 after 48 h incubation (*P*=0.0059, **Figure 3E**); IBV Yamagata virus changed from negative at 0.1 TCID_50_ to detectable with a mean CT value of 33.10 after 24 h incubation (*P*=0.0800) and positive with a mean CT value of 30.39 after 48 h incubation (*P*=0.2333, **Figure 3F**), but this was not statistically significant. In summary, compared with no MTM culturing, 24 h culturing in MTM increased detection sensitivity 10-fold for H1N1, H9N2, and IBVs, 100-fold for H3N2, and 1,000-fold for H7N3. These results demonstrate that MTM treatment can indeed increase the viral load in a sample, thereby lowering the detection threshold when using the same RT-PCR testing kits.

**Table 2:** Initial viral titer (TCID_50_) of the influenza viruses.

Virus type	TCID_50_/0.1ml
H1N1 H9N2 H3N2 H7N3 Yamagata Victoria	2.5×10^-5^ 1×10^-4^ 2.5×10^-5^ 5×10^-7^ 1×10^-5^ 1×10^-4.3^

Using the Reed–Muench method, the viral titers were determined by the 50% tissue culture infectious dose (TCID_50_) based on the cytopathic effect.

**Table 3:** Comparison of the means of the CT values between different MTM incubation time points.

H1N1	0h	24h	*P* value	48h	*P* value	72h	*P* value
100TCID50	28.82 ± 0.14	21.83 ± 1.89	0.0229	24.35 ± 1.40	0.0299	26.51 ± 1.25	0.0834
10TCID50	32.55 ± 0.18	19.37 ± 1.24	0.0025	19.97 ± 0.54	<0.0001	21.81 ± 0.44	<0.0001
1TCID50	39	20.60 ± 0.65	0.0004	18.38 ± 0.13	<0.0001	19.49 ± 0.34	0.0001
0.1TCID50	39	26.26 ± 4.55	0.0399	18.64 ± 1.09	0.0009	21.05 ± 3.59	0.0131
H3N2	0h	24h	*P* value	48h	*P* value	72h	*P* value
100TCID50	39	23.47 ± 1.18	0.0019	21.25 ± 0.34	0.0001	23.95 ± 0.22	<0.0001
10TCID50	39	25.56 ± 0.56	0.0006	21.38 ± 0.56	0.0003	21.91 ± 0.46	0.0002
1TCID50	39	31.42 ± 3.39	0.0605	26.42 ± 2.12	0.0093	22.67 ± 0.028	<0.0001
H9N2	0h	24h	*P* value	48h	*P* value	72h	*P* value
10TCID50	32.63 ± 0.41	30.66 ± 1.19	0.0525	22.12 ± 0.59	<0.0001	20.16 ± 0.23	<0.0001
1TCID50	39	32.49 ± 1.55	0.0184	28.22 ± 2.05	0.0118	22.37 ± 1.49	0.0027
0.1TCID50	39	39	/	33.10 ± 5.83	0.1504	30.94 ± 2.91	0.0135
H7N3	0h	24h	*P* value	48h	*P* value	72h	*P* value
100TCID50	39	20.00 ± 0.64	0.0004	19.64 ± 0.36	0.0001	20.62 ± 0.86	0.0007
10TCID50	39	23.00 ± 0.89	0.0010	19.56 ± 0.37	0.0001	20.63 ± 0.47	0.0002
1TCID50	39	25.47 ± 1.02	0.0019	19.02 ± 0.30	<0.0001	19.95 ± 0.80	<0.0001
0.1TCID50	39	39	/	24.20 ± 3.61	0.0044	21.44 ± 0.68	<0.0001
Victoria	0h	24h	*P* value	48h	*P* value	72h	*P* value
100TCID50	25.49 ± 0.18	20.25 ± 1.12	0.0013	19.79 ± 0.81	0.0003	21.18 ± 0.68	0.0004
10TCID50	29.12 ± 0.80	19.63 ± 0.56	<0.0001	20.70 ± 0.12	0.0024	24.00 ± 7.98	0.3818
1TCID50	33.24 ± 0.43	22.03 ± 1.66	0.0003	20.98 ± 1.73	0.0003	21.99 ± 0.48	<0.0001
0.1TCID50	39	23.51 ± 0.29	0.0001	23.73 ± 2.04	0.0059	21.93 ± 0.47	0.0003
Yamagata	0h	24h	*P* value	48h	*P* value	72h	*P* value
100TCID50	27.55 ± 0.41	24.83 ± 1.13	0.0173	17.64 ± 0.04	0.0005	19.00 ± 0.07	<0.0001
10TCID50	30.05 ± 0.27	28.54 ± 1.91	0.3035	20.21 ± 3.63	0.0418	19.08 ± 3.59	0.0333
1TCID50	32.79 ± 0.52	30.82 ± 3.13	0.3415	27.64 ± 7.18	0.3395	27.55 ± 9.22	0.4284
0.1TCID50	39	33.10 ± 4.30	0.0800	30.39 ± 8.83	0.2333	29.46 ± 10.53	0.2570

CT values that exceed 35 were considered negative, thus they were adjusted to 39 for the purpose of statistical analysis. A t-test was conducted to compare the means of the CT readings at different MTM incubation time points. *P*<0.05 was considered statistically significant difference.

### 3.2 Effect of MTM treatment on long-stored clinical samples containing influenza virus

In total, 19 IAV and 11 IBV clinical samples, which had been stored at −80°C for >1 year, were tested following culture in MTM (**Supplementary Table 1**). **Table 4** shows the RT-PCR results of each sample with and without MTM treatment. The positive detection rate of IAV samples increased from 63.16% (n=12/19) without MTM culturing to 78.95% (n=15/19) after 48 h culturing in MTM (*P*=0.375), and finally reached 89.47% (n=17/19, *P*=0.063) after 72 h culturing, with only two IAV clinical samples remaining undetected, but this was not statistically significant. MTM treatment of IBV clinical samples also increased the positive detection rate from 36.36% (n=4/11, 0 h) to 63.64% (n=7/11, 48 h) (*P*=0.375), and finally 72.73% (8/11, 72 h) (*P*=0.219), with only three IBV samples remaining undetected. These results clearly indicate that culturing with MTM can increase the viral load, augment the detection signal of RT-PCR, and thus effectively decrease the false negative detection rate.

**Table 4:** Comparison of the positive detection rates (%) for IAV and IBV clinical samples cultured in MTM for 0, 48, and 72 h.

Positive rate	0h	48h	*P* value	72h	*P* value
Influenza A	12/19(63.16%)	15/19 (78.95%)	0.375	17/19 (89.47)	0.063
Influenza B	4/11 (36.36%)	7/11 (63.64%)	0.375	8/11 (72.73%)	0.219

A McNemar's test was used to evaluate the percentage of positive detection rates for IAV and IBV clinical samples that were cultivated in MTM for 0, 48, and 72 hours. P<0.05 was considered statistically significant difference.

### 3.3 Clinical performance on the patient samples with MTM treatment

**Figure 4:**
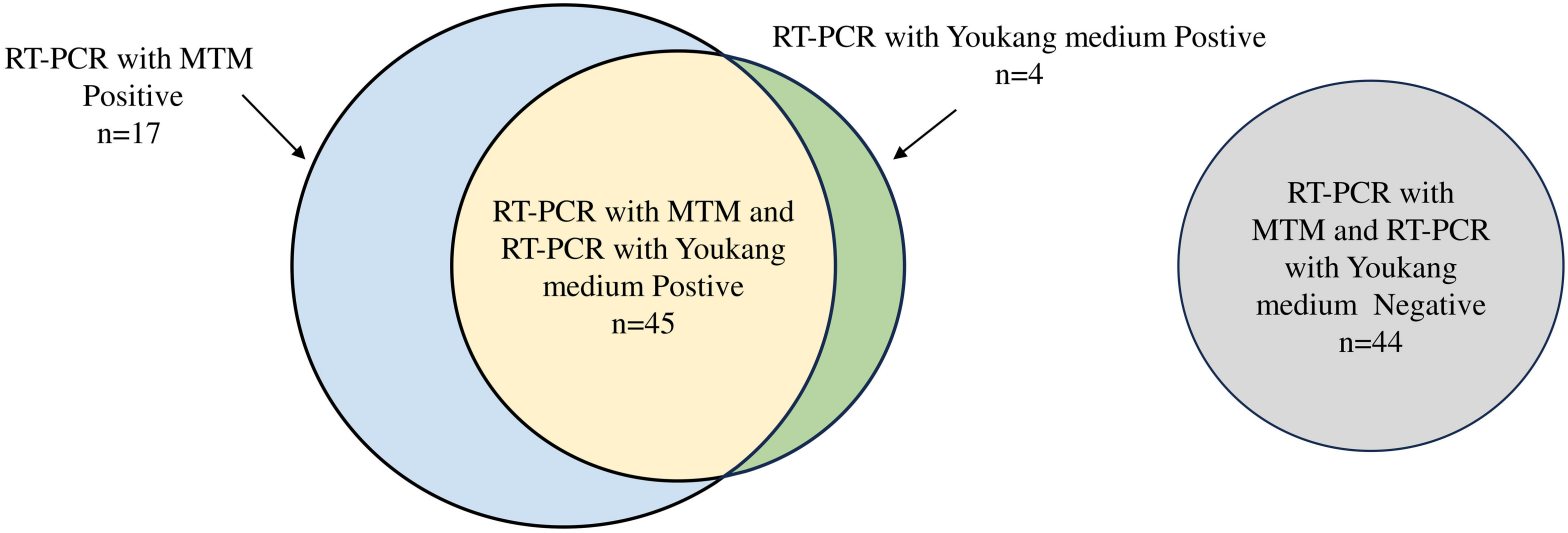
Venn diagram of performance of RT-PCR with MTM versus RT-PCR with Youkang medium. Number of samples positive by RT-PCR with Youkang medium (green), positive by RT-PCR with MTM (blue), or positive by both RT-PCR with Youkang medium and RT-PCR with MTM (yellow). Samples negative for both RT-PCR with Youkang medium and RT-PCR with MTM (grey). Size of circles is proportional to n.

This study included 110 patients, with a median age of 35.00 years (IQR: 21.75–48.00 years) and a male-to-female sex ratio of 1.04. Of the 49 RT-PCR-positive samples, RT-PCR following MTM treatment correctly classified 45 samples as positive for influenza A and four as negative for the virus. RT-PCR following MTM treatment identified 17 additional samples compared with RT-PCR without MTM treatment. RT-PCR following culturing in another transport medium showed positive results in 49 of 110 patient samples (44.55%). By comparison, 62 cases (56.36%) tested positive following MTM treatment, representing an 11.81% rise in the overall positive rate, which was statistically significant (*P*=0.007) (**Figure 4**, **Table 5**). MTM demonstrates the capacity to detect viruses that have been identified as negative by RT-PCR.

**Table 5:** Comparison of the positive detection rates between MTM and Youkang medium.

Method	Positive	Negative	Total	Positive rate	*P* value
Youkang	49	61	110	44.55%	0.007
MTM	62	48	110	56.36%	

A McNemar's test used to compare of the positive detection rates between MTM and Youkang medium. *P*<0.05 was considered statistically significant difference.

The sensitivity of MTM compared with Youkang transport medium was 91.84% (range: 79.52% to 97.35%), and the specificity was 72.13% (range: 58.97% to 82.49%). The positive predictive value was 72.58% (range: 59.56% to 82.78%), and the negative predictive value was 91.67% (range: 79.13% to 97.30%) (**Table 6**).

**Table 6:** The clinical application value of MTM.

Performance	Value	95% Confidence Interval
Sensitivity	91.84%	79.52%~ 97.35%
Specificity	72.13%	58.97%~ 82.49%
PPV (positive predictive value)	72.58%	59.56%~ 82.78%
NPV (negative predictive value)	91.67%	79.13%~ 97.30%
PLR (Positive likelihood ratio)	3.30	2.18~ 4.98
NLR(Negative likelihood ratio)	0.11	0.04~ 0.29
Kappa	0.623	–

Sensitivity = TP/(TP + FN) × 100%, specificity = TN/(TN + FP) × 100%, PPV (positive predictive value) = TP/(TP + FP) × 100%, NPV (negative predictive value) = TN/(TN + FN) × 100%, PLR+(positive likelihood ratio) =sensitivity/(1-specificity), NLR-(negative likelihood ratio)=(1-sensitivity)/specificity, where TP, TN, FP, and FN represent the number of true positives, true negatives, false positives, and false negatives, respectively. The kappa index, which assesses agreement between MTM and Youkang medium, was calculated by Kappa in SPSS 26.0.

## 4 Discussion

Several transport media are available for the transport and storage of virus-containing clinical samples, including phosphate-buffered saline, phosphate-buffered saline-glycerol, brain-heart infusion broth, and universal buffer (Brasel et al., 2015; Zhang et al., 2015). However, these transport media may not effectively preserve clinical samples with low viral loads, and the samples may be degraded (Luinstra et al., 2011; Zhang et al., 2015). Therefore, to increase the true positive results obtained with viral tests, we developed a modified transport medium, MTM, to improve the diagnosis of virus infection and distinguish live from non-live viruses.

We serially diluted stocked IAV subtypes H1N1, H3N2, H7N3, and H9N2, and IBV sublineages Yamagata and Victoria, to compare the detection sensitivities of the diluted viral samples with and without MTM treatment with the same RT-PCR test kit. The kit showed variable levels of sensitivity to different subtypes of influenza, with relatively low sensitivity to H7N3. However, our findings indicated that MTM enhanced the sensitivity of nucleic acid test kits for all target strains. This effect was similar to that of other culturing studies (Abdoli et al., 2013). However, several factors, such as cell volume, cell type, and viral titer can influence the effects on culture (Tian et al., 2012). Thus, we further evaluated the effectiveness of MTM by sensitizing clinical strains of influenza virus subtypes and showed that although there were no significant differences between different culture times, 78.95% of influenza A clinical samples could be cultured, and 63.64% of influenza B clinical samples could be cultured in the medium. However, some clinical specimens were not cultured, possibly owing to the presence of inactivating substances in the viral transport media of some samples. Additionally, a previous study reported that when a large number of cells die, enzymes are released that may partially degrade viral RNA, which may also explain the poor outcomes (Oshiumi et al., 2016).

In our study of clinical samples, RT-PCR following MTM culturing achieved a detection rate of 56.36%, representing a significant improvement of 11.81% over Youkang medium. The clinical diagnostic value of MTM was assessed concurrently using Youkang medium as a baseline reagent and the specificity and positive predictive values of MTM were found to be 72.13% and 72.58%, respectively. We found that some of the samples that tested negative after culturing in Youkang medium (17 out of 61) were effectively cultured using MTM. Overall, we conclude that the transport medium can increase the viral load of these influenza viruses. MTM may therefore be used for the qualitative detection of influenza viruses of very low titer (i.e., the initial viral load cannot be measured). This medium may be suitable for use in central and third-party laboratories for patients with influenza-like illnesses. The medium guarantees the safety of the samples as follows: 1) the tube is made of polypropylene, which does not break easily and ensures that the culture does not leak; 2) this product does not require CO_2_ and can be incubated at 37°C in a constant temperature incubator; and 3) virus lysate or SD lysate can be added to the syringe to ensure virus inactivation and reduce the risk of sample leakage due to cap opening and thus is useful for communal public health testing of respiratory outbreaks and diagnosing individual patients.

Our study had some limitations. Temperatures <37°C affect the cultivation effect of the medium, but it can be used as an ordinary transport medium under such conditions. Additionally, the relatively long incubation time may be more suitable for hospitalized patients with low viral loads who do not test positive by routine PCR. Our clinical study found that four RT-PCR-positive samples tested negative following MTM treatment; therefore, we suggest that paired samples are taken from patients with severe disease. If the transport medium is determined to be negative in the first detection and the clinician is highly suspicious, the MTM can be maintained at 37°C for 24–48 h before undergoing additional testing, which may offer significant diagnostic results.

## Data availability statement

The original contributions presented in the study are included in the article/**Supplementary Material**. Further inquiries can be directed to the corresponding authors.

## Ethics statement

The studies involving humans were approved by The Ethics Committee of The First Affiliated Hospital of Guangzhou Medical University. The studies were conducted in accordance with the local legislation and institutional requirements. The participants provided their written informed consent to participate in this study.

## Author contributions

ZZ: Writing – original draft, Writing – review & editing. QL: Writing – original draft, Writing – review & editing. HG: Funding acquisition, Methodology, Validation, Writing – review & editing. YoL: Investigation, Resources, Writing – review & editing. LL: Formal Analysis, Investigation, Validation, Writing – review & editing. YuL: Formal Analysis, Writing – review & editing. YF: Methodology, Writing – review & editing. LL: Formal Analysis, Investigation, Writing – review & editing. QX: Investigation, Validation, Writing – review & editing. YZ: Investigation, Methodology, Resources, Writing – review & editing. ZL: Methodology, Writing – review & editing. WG: Investigation, Resources, Writing – review & editing. ZY: Funding acquisition, Investigation, Resources, Writing – review & editing.
