# Supplementary material for: Modified transport medium for improving influenza virus detection
Source: Front Cell Infect Microbiol. 2024 Jul 4;14:1399782. doi: 10.3389/fcimb.2024.1399782 (PMC11254831; doi:10.3389/fcimb.2024.1399782)
Supplement: Supplementary file 1 [file Table_1.docx]

**Supplementary Material**

Supplementary Table 1

| **Influenza A** | | | |
| --- | --- | --- | --- |
| **Specimen number** | **0h CT values** | **48h CT values** | **72h CT values** |
| **A1** | **NA (36.27)** | **29.01** | **29.01** |
| **A2** | **25.09** | **18.26** | **21.02** |
| **A3** | **NA (36.41)** | **29.93** | **31.24** |
| **A4** | **33.76** | **28.41** | **30.25** |
| **A5** | **NA** | **34.50** | **29.87** |
| **A6** | **34.77** | **18.41** | **30.87** |
| **A7** | **34.58** | **21.38** | **20.22** |
| **A8** | **NA** | **NA** | **NA** |
| **A9** | **NA (38.58)** | **NA (35.05)** | **34.63** |
| **A10** | **31.52** | **26.24** | **18.69** |
| **A11** | **NA (38.68)** | **NA** | **NA** |
| **A12** | **33.42** | **NA (35.57)** | **34.27** |
| **A13** | **30.69** | **31.09** | **28.92** |
| **A14** | **24.54** | **22.05** | **18.08** |
| **A15** | **27.80** | **26.58** | **21.73** |
| **A16** | **NA (38.42)** | **33.71** | **34.70** |
| **A17** | **23.66** | **22.84** | **20.18** |
| **A18** | **28.25** | **28.28** | **27.21** |
| **A19** | **23.43** | **20.83** | **21.73** |
| **Proportion of positive detection (%)** | **12/19 (63.16)** | **15/19 (78.95)** | **17/19 (89.47)** |

| **Influenza B** | | | |
| --- | --- | --- | --- |
| **Specimen number** | **0h** | **48h** | **72h** |
| **B1** | **33.54** | **34.89** | **34.97** |
| **B2** | **NA (36.29)** | **19.69** | **18.71** |
| **B3** | **33.23** | **34.11** | **34.16** |
| **B4** | **NA** | **NA (35.88)** | **NA (37.08)** |
| **B5** | **NA (35.24)** | **25.54** | **21.86** |
| **B6** | **NA (37.90)** | **32.97** | **31.69** |
| **B7** | **NA (36.74)** | **21.40** | **19.67** |
| **B8** | **34.53** | **NA (35.30)** | **NA (35.46)** |
| **B9** | **NA** | **NA (37.45)** | **34.24** |
| **B10** | **30.30** | **23.79** | **21.01** |
| **B11** | **NA (35.59)** | **NA (36.54)** | **NA (36.67)** |
| **Proportion of positive detection (%)** | **4/11 (36.36)** | **7/11 (63.64)** | **8/11 (72.73)** |

NA: Negative; The CT cut-off value for determining a positive sample of the RT-PCR kit was ≤ 35 CT. Thus, CT values > 35 is considered as negative;
